# Elaboration and Characterization of Conductive Polymer Nanocomposites with Potential Use as Electrically Driven Membranes

**DOI:** 10.3390/polym11071180

**Published:** 2019-07-13

**Authors:** Leire Sangroniz, Ainara Sangroniz, Mercedes Fernández, Agustin Etxeberria, Alejandro J. Müller, Antxon Santamaria

**Affiliations:** 1 POLYMAT and Polymer Science and Technology Department, Faculty of Chemistry, University of the Basque Country UPV/EHU, 20018 San Sebastian, Spain; 2 IKERBASQUE, Basque Foundation for Science, 48013 Bilbao, Spain

**Keywords:** nanocomposites, recycling, stimuli responsive, Joule effect, permeability

## Abstract

In this work, a general, facile, and relatively low-cost method to produce electrically driven non-porous membranes by revalorization of recycled polyolefins is proposed. The polymer matrices are poly(propylene) (PP) and poly(ethylene) (PE) and their corresponding recycled samples, which are respectively mixed with carbon nanotubes (CNT). The performances of the elaborated nanocomposites are studied by morphological, rheological, and electrical conductivity tests. The Joule heating effect is evaluated by applying an electric field and recording the corresponding temperature rise. An increase of 90 °C is obtained in certain cases, which represents the highest temperature enhancement reached so far by the Joule effect in thermoplastics, to our knowledge. The work shows a route to develop stimulus (voltage)-response (temperature) materials with low cost and with potential applications in many fields. As an example, the increase of the permeability with temperature of membranes made of the indicated nanocomposites, is analyzed.

## 1. Introduction

Stimuli-responsive materials have gained an increasing interest in the last years due to their promising applications. These materials are able to change their physical or chemical properties under external stimuli such as light, pH, electric current, magnetic field, or heat, among others. In the case of polymers, such stimuli can change their shape, chain dimension, solubility, or their secondary interactions, as has been reported in the literature [1,2].

Most of previous studies are focused on the search of materials that can react to biological stimuli, with the aim of creating new diagnosis techniques and drug delivery devices. On the other hand, there are also studies on conceiving materials that can change their shape under appropriate stimuli, like heat, electric, or magnetic field, among others [1,2]. These materials can have potential applications in biomedicine and also in engineering applications.

Apart from the aforementioned applications, interesting and promising advances have been carried out in the field of stimuli responsive membranes. Different kinds of membranes have been studied: Porous membranes used in filtration processes, as well as non-porous membranes [3,4,5].

Membranes that are able to react under electromagnetic field have been developed to regulate the concentration of solvents, as well as in controlled release of active molecules, water purification, separation processes, and sensors [3,4,5]. In the case of membranes susceptible to react to electric stimulus, only porous membranes have been studied, focusing on the size change of the pores with the electric field. Regarding the use of Joule heating effect, so far only a couple of works have been reported in the literature, both using porous membranes and involving pervaporation processes [6,7]. In particular, a polyvinyl alcohol/CNT film was obtained and deposited on a porous membrane substrate, to be heated through an electric current and, so, increase the evaporated amount of water [6].

Within the context of electrically activated membranes, in this work, we focus on the development of stimuli responsive non-porous membranes based on commodity polymers filled with carbon nanotubes (CNT), which allows obtaining electrically conductive nanocomposites. As opposed to porous membranes made of polymer gels sensible to electric fields and films deposited on membranes, we propose to take advantage of the Joule effect to control the temperature of non-porous nanocomposite membranes. Actually, the use of conductive nanoparticles allows obtaining stimuli responsive non-porous membranes that encompass a wide range of properties and applications (drug delivery, solvents concentration control, etc.), since different polymer matrices can be considered.

Although a lot of research is focused on promising applications in the area of polymers, the environmental issues should not be neglected. It should be taken into account that nowadays a large amount of plastic waste is generated from the use of polymers in a wide range of products. In 2016 27.1 million tons of plastic waste was generated: 31.1% was recycled, 41.6% was recovered by energy recovering, whereas 27.3% went to landfills [8]. This reflects the necessity of polymer recycling, which is an economical option. There are several examples of polymer that are recycled in industry such as PET, polyamides, or polyolefins. Recycled PET is used to obtain films or fibers and polyamides are also converted in fibers. Regarding recycled polyolefins, they can be employed to obtain pipes, vehicle parts, or household goods, among others [9].

In general, the mechanical recycling of the materials results in products with relatively poor properties in comparison with the original ones, because the material is submitted to high temperature and shear in which degradation occurs. In order to obtain recycled materials with improved properties, nanoparticles can be used. The use of conductive nanoparticles, such as carbon nanotubes, would allow the revalorization of the recycled polymers, since the presence of carbon nanotubes can result in semiconductive materials.

In this work, nanocomposites based on neat and recycled PP and PE, as well as membranes elaborated from these materials, are studied. The aim is to take advantage of the Joule heating effect to control the permeability of the membranes. For that, first a physical characterization of the different nanocomposites is performed employing transmission electron microscopy (TEM), rheological techniques, and conductivity measurements. Then, the Joule heating effect of the different samples is analyzed, and the permeability of the samples is determined.

## 2. Materials and Methods

### 2.1. Materials, Nanocomposites, and Membranes Preparation

Polypropylene (PP) (ISPLEN PP070G2M) was supplied by Repsol (Madrid, Spain) whereas high-density polyethylene (PE) (Rigidex, HD6070EA) was obtained from INEOS (Zierbena, Spain). The recycled polymers (Rec-PP) and (Rec-PE) were provided by Suez (Geleen, The Netherlands). Carbon nanotubes in the form of powder were supplied by Cheap Tubes Inc. (Grafton, Vermont, USA) with a diameter of D = 30–50 nm and length of L = 10–20 µm. The nanocomposites were prepared in the molten state using a Collins co-rotating twin screw extruder. The PP/CNT were prepared at 210–230 °C whereas PE/CNT nanocomposites were mixed at 180–200 °C at 150 rpm. In all the cases, the nanofiller content was 5% in weight. The membranes were prepared by hot pressing at 180 and 160 °C for PP and PE, respectively. The films were dried in an oven at 70 °C for 24 h in vacuum and for at least 6 days at room temperature under vacuum.

### 2.2. Characterization Techniques

The dispersion of carbon nanotubes was analyzed by transmission electron microscopy (TEM; TECNAI G2 20 TWIN (FEI), Hillsboro, Oregon, USA) amploying an acceleration voltage of 200 keV. The samples were prior cut by ultramicrotomy (Leica EMFC 6, Wetzlar, Germany). Thermal properties were studied employing a differential scanning calorimetry (DSC, Perkin Elmer, Waltham, MA, USA) and the measurements were performed under nitrogen atmosphere from 0 °C to 200 °C at 20 °C min^-1^. In order to study the rheological properties, small amplitude oscillatory shear measurements were carried out using a rheometer (ARG2, TA Instrument, New Castle, DE, USA) with parallel plates under nitrogen atmosphere at 180 °C for PP and 160 °C in the case of PE. Conductivity measurements were carried out employing the dielectric analysis option (DETA) of the ARES Rheometer (TA Instrument, New Castle, DE, USA) which is coupled to a Novocontrol (Madrid, Spain) interface. The measurements were performed at room temperature in a frequency range of 10^2^ to 10^7^ Hz. To analyze the surface heating effect of the sample applying an electric voltage (Joule effect), the temperature was recorded using a FLIR infrared camera (Wilsonville, OR, USA) in home-made equipment. The samples were prepared by compression molding at the adequate temperature for each sample and cooling down using a water-ice bath. The dimensions of the analyzed samples were 26 mm × 11 mm × 1.0 mm. Permeability to water vapor rate was measured monitoring the weight loss according to ASTM E96-95 standard test method [10]. Oxygen permeability was measured in MOCON OX-TRAN equipment (Minneapolis, MN, USA) at 23 °C and in dry conditions [10]. Films in the range of 90–160 m thickness have been measured. The reported values are at least the average of 3 measurements.

## 3. Results

### 3.1. Morphology and Dispersion of Nanofillers

**Figure 1:**
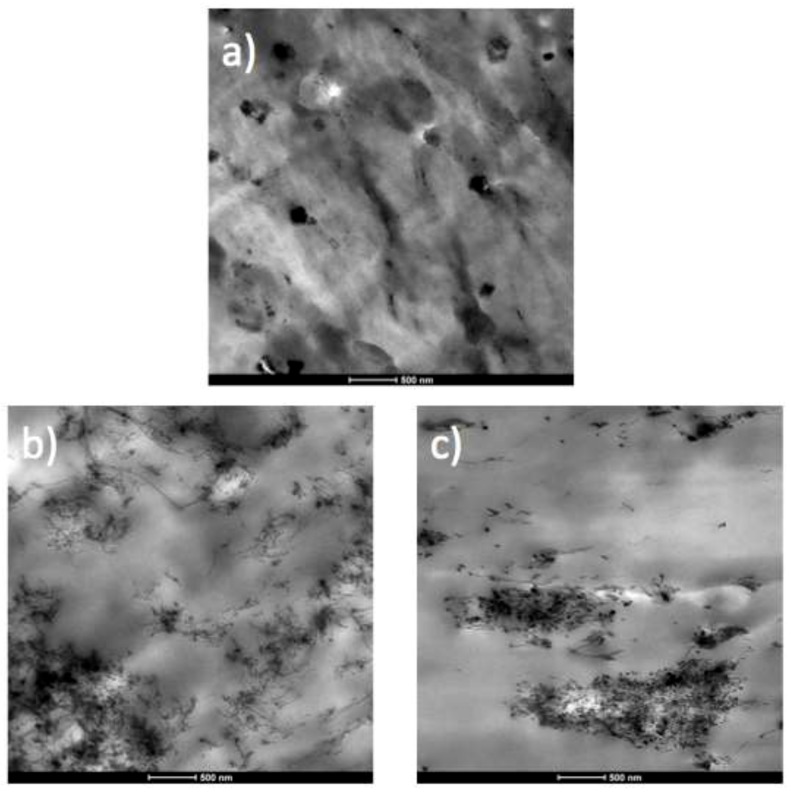
TEM images of the recycled poly(propylene) (PP) and the different polymer/carbon nanotubes (CNT) nanocomposites. (**a**) Rec-PP, (**b**) PP/CNT and (**c**) Rec-PP/CNT.

In order to analyze the distribution of CNTs in the matrix transmission electron microscopy (TEM) was employed; micrographs of PP-based nanocomposites are displayed in Figure 1 and those of the PE-based nanocomposites are shown in Supporting Information, Figure S1. In the case of recycled PP without CNTs, it can be observed that there is a second phase that forms droplets in the PP matrix. The second phase is constituted by poly(ethylene), because although during recycling both polyolefins are separated, some impurities can remain. This is corroborated by differential scanning calorimetry (DSC) results, as it will be discussed in the next section. Furthermore, some nanoparticles can be observed. To determine the nature of the nanoparticles, the polymer has been carbonized by submitting at high temperatures and the residue has been characterized by Infra-red (IR) spectroscopy. According to IR data (please see Figure S2 in Supporting Information), the residue contains titanium dioxide (rutile type) and some traces of calcium carbonate and talc. TGA measurements reveal that there is 1.5% of inorganic nanoparticles in weight (Figure S3). Regarding nanocomposites, it can be stated that in the case of PP matrix, the CNTs were better distributed in commercial PP than in the recycled PP. In the case of recycled PP, it is observed that nanoparticles are agglomerated inside the dispersed phase. A similar result is obtained for PE-based nanocomposites.

### 3.2. Thermal Properties

**Figure 2:**
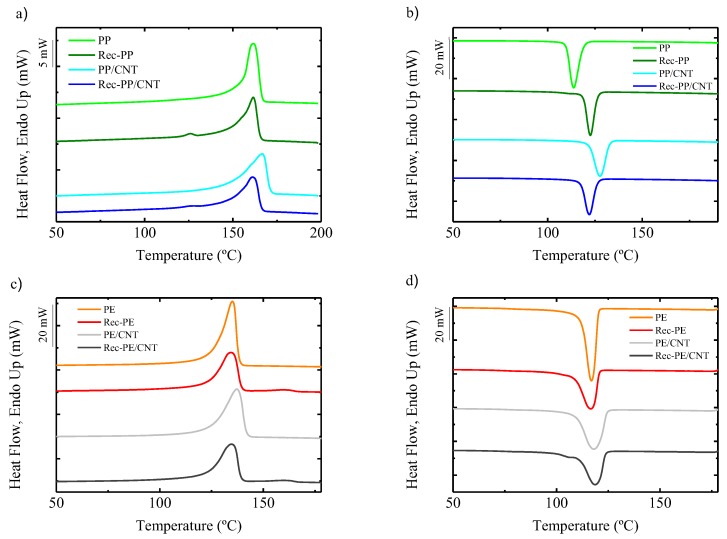
DSC heating and cooling scans of all the samples studied in this work: (**a**) heating and (**b**) cooling scans of PP based samples, (**c**) heating and (**d**) cooling scans of PE based materials.

The thermal properties of the commercial and recycled homopolymers and nanocomposites have been studied; the results are shown in Figure 2. For both commercial and recycled PP, a melting peak at 161.9 °C is observed. In the case of recycled PP, an additional melting peak appears at about 125.6 °C, which corresponds to the melting of the aforementioned PE dispersed phase. This indicates that during recycling, the separation of both polyolefins is not complete, and some traces of the other component remain. The melting peak of PP/CNT nanocomposite is increased 4 °C with respect to pure PP, but in the case of Rec-PP/CNT, no such enhancement is observed.

Regarding PE-based samples, commercial PE shows a melting peak at 135.1 °C. Rec-PE shows two melting peaks, one similar to commercial PE and the other at 160.6 °C, which corresponds to the traces of PP. The presence of CNTs increases the melting temperature 2 °C in the case of commercial PE. Nevertheless, Rec-PE/CNT shows a similar T_m_ to that of Rec-PE and PE. It also shows a second melting point at 160.9 °C due to the presence of PP.

Cooling DSC scans of PP-based materials show only the peak corresponding to the crystallization of the PP at 113.6 °C. The recycled PP shows a higher T_c_ value, 122.5 °C, which is compatible with the presence of inorganic nanoparticles mentioned before, i.e., titanium dioxide, calcium carbonate, and talc, that can act as nucleating agents. The addition of CNTs to PP increases the crystallization temperature significantly, which reflects the nucleating effect of those nanoparticles. But, in the case of Rec-PP/CNT nanocomposite, the T_c_ is similar to that of recycled PP, apparently because the impurities present are also able to nucleate and compete with CNTs, rendering their nucleation efficiency lower in comparison to the case of neat PP.

Regarding the cooling DSC scans of PE-based materials, an increase of 2 °C is observed for PE/CNT. For the rest of the samples the differences are within the error of the experimental technique.

### 3.3. Rheological Properties

**Figure 3:**
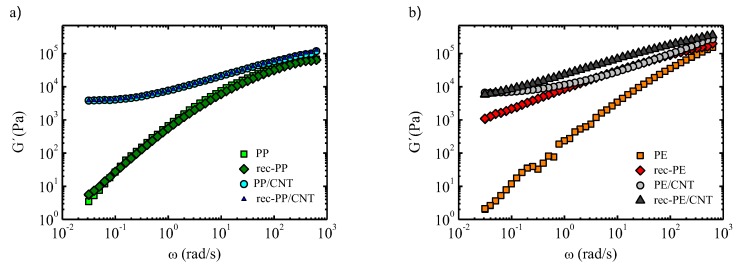
Elastic moduli against frequency for the different systems studied in this work: (**a**) PP based systems and (**b**) PE based materials.

The rheological measurements for the different nanocomposites were performed using small amplitude oscillatory shear (SAOS) measurements in the linear regime. Figure 3 shows the elastic moduli obtained by frequency sweeps for neat polymers and nanocomposites. In the case of PP, it can be observed that the elastic moduli of PP and recycled PP overlap. Thus, the molecular weight distributions of both commercial and recycled PP are quite similar and, on the other hand, there is not a noticeable effect of the small number of nanoparticles contained in the recycled sample on the chain dynamics in the terminal zone. When CNTs are added, the storage modulus, *G’*, levels off at low frequencies and overcomes the loss modulus, *G”*, (not shown in the plot) (*G´* > *G”*), which stands for a suppression of the flow or terminal viscoelastic zone. This is compatible with the formation of a percolated network, which results from nanofiller–nanofiller and nanofiller–polymer interactions and hinders the motion of the polymer chain as a whole [11,12,13]. It is interesting to note that the nanocomposites obtained from commercial PP and recycled PP show practically the same G´ value, which means that the dispersion of CNTs is analogous in both cases.

Regarding the PE system, commercial and recycled PE show quite different rheological behavior. The elastic modulus, G’, of recycled PE is considerably higher than that of the original polymer, almost 3 orders of magnitude. This arises from the possible contamination with PP and LDPE traces, that bring about a more elastic behavior. As could be expected, the addition of CNTs provokes a solid-like behavior, being G´ > G” in the terminal zone, due to the formation of a percolated network, similarly to the results obtained with PP.

### 3.4. Electrical Conductivity

**Figure 4:**
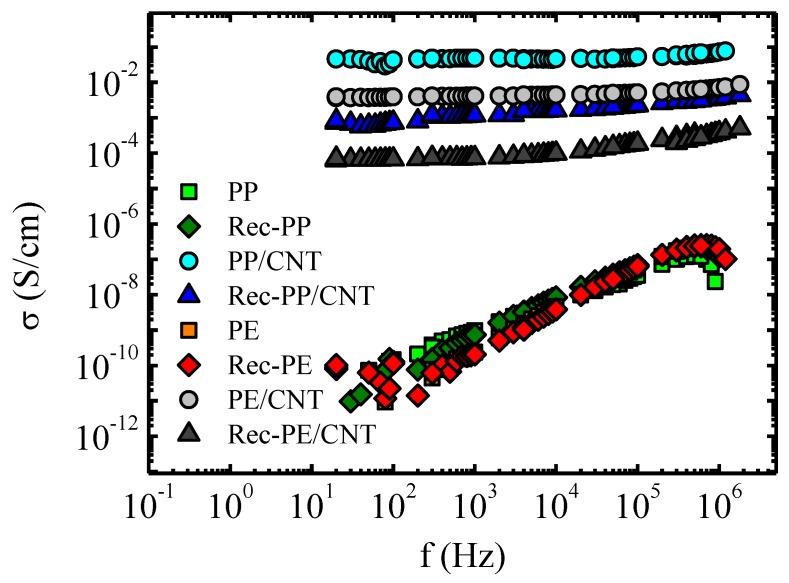
Electrical conductivity as a function of frequency for PP- and poly(ethylene) (PE)-based samples at room temperature.

The results of the electrical conductivity at room temperature are shown in Figure 4. Commercial, as well as, recycled PP and PE showed the typical insulating behavior of polymers, being the value of conductivity 10^−11^ S m^−1^ at low frequencies and rising up to 10^−7^ S m^−1^ at high frequencies. For the nanocomposites, the electrical conductivity was practically independent of frequency, which is a symptom of electronic conductivity [12]. The invariance of conductivity with frequency also indicates that the electrical percolation threshold of the CNTs has been reached. Higher conductivity values were found for PP/CNT nanocomposite than for Rec-PP/CNT nanocomposite: 0.1 S m^−1^ face to 1 × 10^−3^ S m^−1^. This reflects that the presence of PE impurities and insulator nanoparticles (like TiO_2_) hinder the conductivity of the material. In terms of comparison, Huegun et al. [14] reported 1 × 10^−2^ S m^−1^ using CNT powder to elaborate a PP/5% CNT nanocomposite and Pöschke et al. [15] obtained a value of 0.2 S m^−1^ with masterbatch CNT. Figure 4 shows that nanocomposites based on PE brought about lower electrical conductivity values than nanocomposites based on PP. At the lowest frequency conductivities are: 1 × 10^−2^ S m^−1^ for the nanocomposite prepared with commercial PE and 1 × 10^−4^ S m^−1^ for the nanocomposite prepared with recycled PE. In the literature, a value of 3 × 10^−2^ S m^−1^ has been reported for high density poly(ethylene) (HDPE) with MWCNT (nanofiller content of 5%), which is quite similar to the value obtained in this work [16].

### 3.5. Joule Heating Effect

Taking advantage of the Joule effect, the electric heating of the samples was studied by applying different voltages and monitoring the corresponding temperature increase by means of an infrared camera.

**Figure 5:**
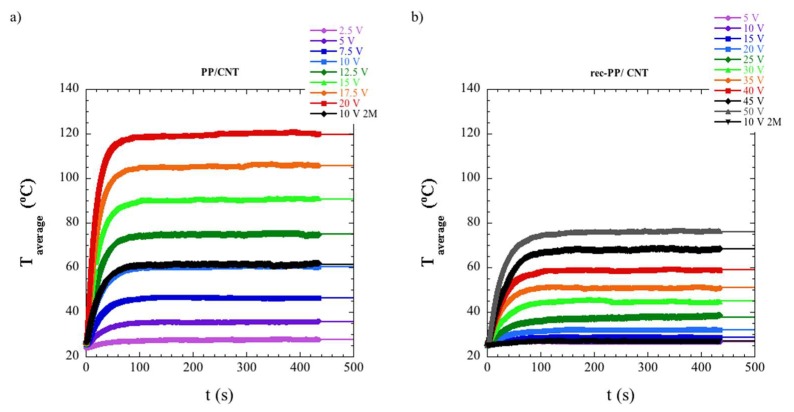

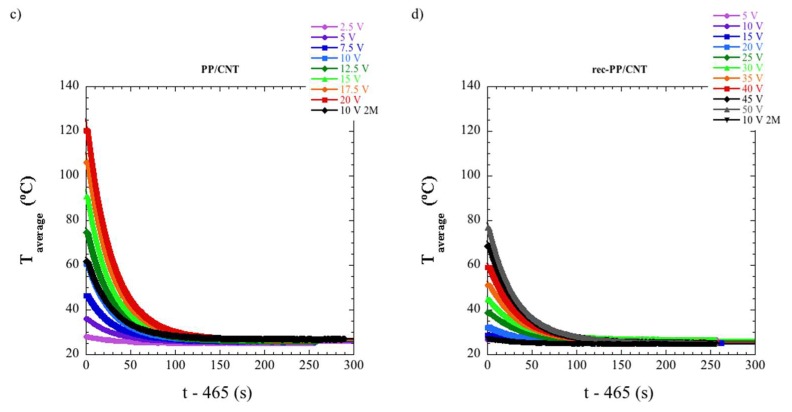
The heating and cooling step of the Joule heating effect is shown for PP nanocomposites. The data are fitted to Equation (1) in the case of the heating step and to Equation (3) for the cooling process. (**a**) Heating of PP/CNT nanocomposite, (**b**) heating of recycled polymers (Rec-PP)/CNT nanocomposite, (**c**) cooling of PP/CNT nanocomposite, and (**d**) cooling of Rec-PP/CNT nanocomposite. 10 V 2M stands for a second measurement carried out applying 10 V after a first run, to test reproducibility.

Both poly(propylene) based nanocomposites, i.e., the sample prepared using commercial PP and that prepared with recycled PP, showed a significant increase of temperature applying an electric voltage, as can be seen in Figure 5. The results corresponding to PE nanocomposites are shown in Supporting Information (Figure S4). The increase of temperature is lower for the PE/CNT nanocomposite than for the PP-based nanocomposites. For the nanocomposite based on recycled PE, Rec-PE/CNT, the temperature does not increase at all, even at the highest applied voltage. These results could be expected, in view of the electrical conductivity results shown in Figure 4.

The temperature of the sample was stabilized after a time of approximately 150 s. When the voltage was switched off, the temperature decreased to the initial temperature in approximately 120 s. Hence, the cooling process was faster than the heating process.

The analysis of the Joule effect was divided in three steps: a) Heating of the sample, b) period in which temperature is stabilized at maximum value, and c) cooling of the sample, once the voltage is removed. Each step can be fitted to the following respective equations [17,18,19],

(1) $$\mathrm{Heating}\;\mathrm{regime}:\;T_{t}=\left(T_{max}-T_{0}\right)\;\left(1-e^{-t/\tau_{h}}\right)+T_{0}$$

(2) $$\mathrm{Maximum}\;\mathrm{temperature}\;\mathrm{regime}:\;h_{r+c}=\frac{I_{c}V_{0}}{T_{m}-T_{0}}$$

(3) $$\mathrm{Cooling}\;\mathrm{regime}:\;T_{t}=\left(T_{max}-T_{0}\right)\;\left(e^{-t/\tau_{c}}\right)+T_{0}$$

where *t* is the time, *T*_max_ is the maximum temperature, *T*_0_ is the initial temperature, *T*_t_ is the temperature at each time, *τ*_h_ and *τ*_c_ are a characteristic time, during heating and cooling, respectively, *h*_r+c_ is the heat transferred by radiation and convection, and *I*_c_ and *V*_0_ are the current and applied voltage values, respectively.

The corresponding values of the fitting parameters of Equations (1)–(3) are shown in Tables S1–S3 of supporting information. The lowest value of the heat transferred by radiation and convection *h*_r+c_ is shown by PP/CNT nanocomposite, followed by PE/CNT nanocomposite, whereas the sample with the highest *h*_r+c_ value is Rec-PP/CNT nanocomposite. On the other hand, it can be observed that, *τ*_h_, i.e., the heating characteristic time, is reduced with the applied voltage. This apparently contradicts the results obtained by Jeong et al. [18], since these authors reported that *τ*_h_ is independent of the applied voltage for an epoxy cured/graphene nanocomposite with 5% graphene, which requires a very short time, 6 s, to reach the maximum temperature. Probably a very brief heating time impedes an accurate analysis of the effect of voltage on *τ*_h_. On the other hand, it can be observed that the characteristic time follows this trend: PP/CNT < PE/CNT < Rec-PP/CNT. No significant differences are observed in the characteristic times during heating and cooling.

**Figure 6:**
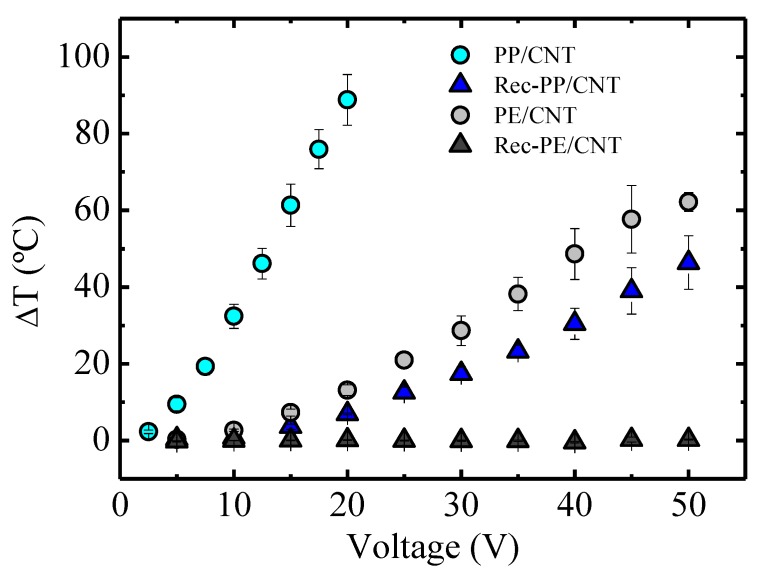
Increase of the temperature as a function of the applied voltage for the different nanocomposites.

The results of the effect of voltage on stabilized temperature for the four nanocomposites are presented in Figure 6. As an example, in the case of the PP/CNT nanocomposite, a voltage of 20 V provoked an increase of 90 °C. This is an outstanding temperature enhancement, never reached before by Joule effect in thermoplastic nanocomposites, as far as we are aware. In the literature, a temperature increase of 20 °C was reported for a UV cured epoxy/graphene nanocomposite with 2% of graphene applying 20 V [20], whereas for a poly(ether-urethane)/carbon nanotube nanocomposite, the temperature increased 50 °C when applying a voltage of 25 V [21].

Applying the same voltage (20 V) to the nanocomposite based on recycled PP, Rec-PP/CNT, induced only a 7 °C increase, due to the lower electrical conductivity of this system. In turn, this is a consequence of the presence of PE phase and other nanoparticles, such as TiO_2_, that disrupt the conducting path of the carbon nanotubes.

As it was expected, considering rheological and conductivity results, the temperature enhancements reached with PE-based nanocomposites were more modest. Only the nanocomposite based on commercial PE gave rise to a significant temperature increase, about 14 °C with respect to room temperature, applying a voltage of 20 V. This low temperature increase is related to the low conductivity of this material. Regarding the recycled PE based nanocomposite, it did not show Joule heating effect. This suggests that electrical conductivity values of about 10^−4^ S m^−1^ (Figure 4) are insufficient to be effective for the purposes involved in the stimuli-responsive materials of this paper.

### 3.6. Permeability

#### 3.6.1. Water Vapor Transmission Rate

**Figure 7:**
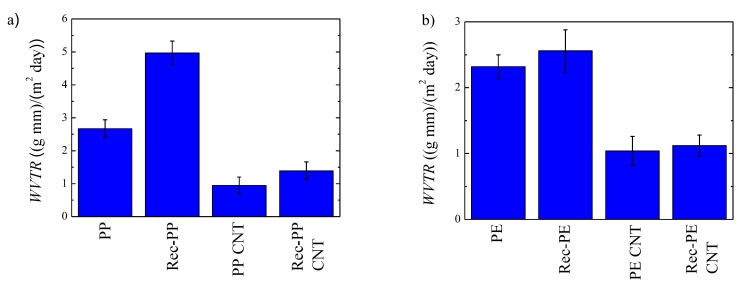
Water vapor transmission rate for (**a**) systems based on PP and (**b**) systems based on PE.

Water vapor transmission rate (WVTR) was measured for all the samples at 25 °C, see Figure 7. Poly(propylene) and recycled poly(propylene) show a value of 2.67 and 4.50 (g mm)/(m^2^ day), respectively. It is worthy to note the higher permeability obtained for the recycled PP. As mentioned previously, from FTIR analysis, it has been concluded that recycled PP contains TiO_2_ and traces of calcium carbonate and talc. Furthermore, DSC and TEM analysis show that there is a small amount of poly(ethylene). Taking into account that PP is immiscible with PE [22], it can be concluded that PE droplets may have a poor adhesion enhancing the diffusion of the penetrant. The presence of the nanoparticles should decrease the permeability since impermeable particles create a tortuous pathway decreasing the diffusion coefficient and therefore the permeability [23,24]. However, the obtained results indicate that the presence of PE droplets overcomes this effect. Crystallinity is another factor that plays an important role in permeability, since crystallites are considered impermeable and they act as non-permeable particles [25,26]. In this case, no significant differences can be observed in crystallinity level (Table S4 in Supporting Information).

The addition of carbon nanotubes leads to a significant improvement on the barrier character of both commercial and recycled PP, with a reduction of up to 70%. The reduction of the permeability arises from the presence of impermeable carbon nanotubes that create a tortuous pathway, as mentioned previously, reducing the permeability [23,24]. The presence of nanotubes does not significantly change the crystallinity level of the samples. The dispersion of nanoparticles plays an important role on the permeability; in this case, a good dispersion is obtained for PP, but agglomerates are formed for recycled PP. However, from the obtained results, it can be concluded that this poor dispersion has no negative effect on the permeability.

In Figure 7b, the water vapor transmission rate of systems based on poly(ethylene) are shown. Poly(ethylene) and recycled poly(ethylene) show a value of 2.32 and 2.56 (g mm)/(m^2^ day), respectively. Again, a higher value is obtained for recycled PE. Rec-PE, as well as Rec-PP, both contain TiO_2_ and traces of calcium carbonate and talc and there is a small amount of PP. The poor adhesion between PE and PP phases [22] could facilitate the permeation of the penetrants. Furthermore, the higher permeability obtained for recycled PE, as compared to pristine PE, arises also from the lower crystallinity level, (Table S4 in Supporting Information). As mentioned previously, the crystallites are considered impermeable and create a tortuous pathway [25,26].

The incorporation of CNTs decreases considerably the permeability giving rise to much lower values: 1.04 and 1.12 (g mm)/(m^2^ day), for PE/CNT and Rec-PE/CNT, respectively. This is a priori the expected behavior, since, as mentioned previously, the nanoparticles are impermeable and they create a tortuous pathway decreasing the diffusion and therefore the permeability [23,24]. Comparing PP and PE polymers it can be observed that the incorporation of CNT to PP matrix decreases more severely the permeability.

#### 3.6.2. Oxygen Permeability

**Figure 8:**
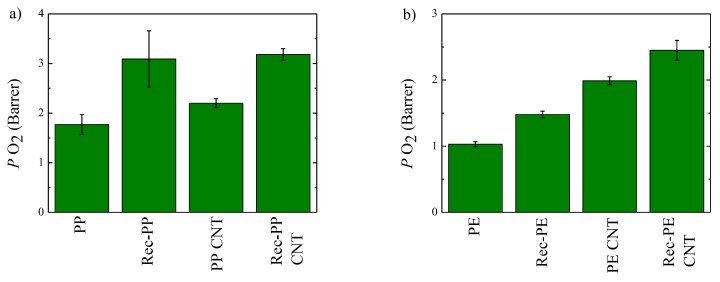
Oxygen permeability for: (**a**) Systems based on PE and (**b**) systems based on PP.

Oxygen permeability has been measured for different PP- and PE-based samples. The obtained results are shown in Figure 8. A value of 1.77 Barrer is obtained for poly(propylene and 3.09 Barrer for the recycled PP. As mentioned previously the higher permeability of the recycled material arises from the presence of PE droplets that are not compatible with PP [22] and may have a poor adhesion between them.

The incorporation of CNT significantly increases the permeability of both PP and recycled PP. These results indicate that although the nanotubes can create a tortuous pathway, they also create a preferential channel where the permeation of penetrant can be enhanced [24,27].

These results contradict those obtained for the water vapor transmission rate, where permeability is decreased with the incorporation of nanotubes. This can be explained by the chemical nature of the penetrants: Water vapor can interact strongly through hydrogen bonds and dipole–dipole interactions whereas oxygen can form only weak Van der Waals interactions [24]. Bearing this in mind, it is suggested that water molecules interact with both, the polymer matrix and CNT nanoparticles, reducing the diffusion coefficient. Furthermore, water can also form clusters that would lessen the diffusion coefficient [28]. Certainly, oxygen is less interacting than water and, therefore, no such reduction is observed with the incorporation of CNT nanoparticles.

The permeabilities to oxygen of PE and recycled PE are, respectively, 1.03 and 1.48 Barrer. As mentioned previously, this higher value can be related to the presence of PP. The incorporation of CNT to both samples leads to an increase of the oxygen permeability. This result is similar to that obtained for PP and the differences observed between water vapor and oxygen can be attributed to the aforementioned different chemical nature of the penetrants. The obtained results suggest that in the nanocomposites, water could be more prone to form clusters hindering the diffusion and therefore decreasing the permeability [28].

#### 3.6.3. Prediction of the Permeability

In literature different approaches have been carried out to predict the permeability of nanocomposites [29,30] and in this work some of these models have been applied for the case of water.

Different models such as Nielsen, Cusller random and regular array, Gusev–Lusti, Fredrickson–Bicerano, and Bharadwaj [29,30] models have been applied, (Table S6 in Supporting Information). In our case, the models were modified to take into account also the presence of TiO_2_, besides to CNT, but it was observed that the difference was irrelevant.

Almost all the models overestimate the permeability of the nanocomposite, being Cussler random and Fredrickson–Bicerano models the ones that show the best results for PP/CNT and Rec-PP/CNT, as can be seen in Table 1.

In the case of PE nanocomposites, Cussler and Fredrickson–Bicerano models predict quite well the permeability of rec-PE/CNT. However, in the case of PE/CNT, these models underestimate the experimental values and Nielsen and Gusev–Lusti models are more adequate in this case.

#### 3.6.4. Effect of Temperature on Permeability

**Figure 9:**
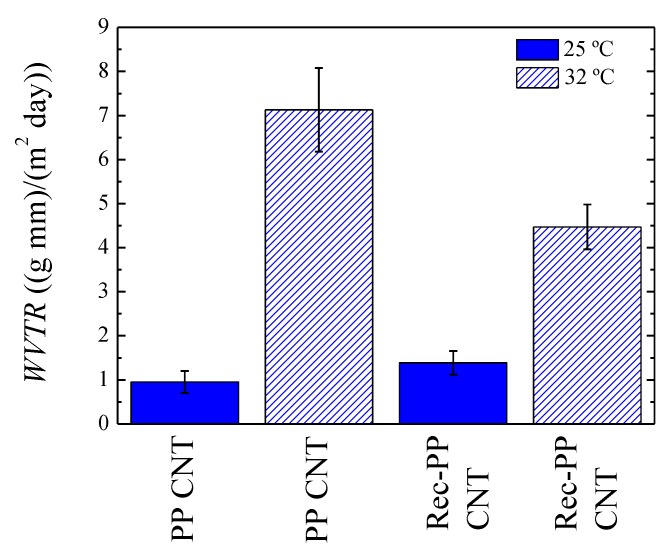
Water vapor transmission rate for PP/CNT and rec-PP/CNT at 25 and 32 °C.

It is known that temperature affects permeability of polymers and in our case, PP-based nanocomposites have been considered, because, as can be seen in Figure 6, a significant Joule effect is observed for these samples. In particular, the results at 25 and 32 °C of the permeabilities of the membranes elaborated with PP/CNT and Rec-PP/CNT nanocomposites are presented in Figure 9. We remark that for PP/CNT, the water vapor transmission rate is increased approximately seven times reaching a value of 7.13 ((g mm)/(m^2^ day)) at 32 °C.

Nevertheless, it has to be recalled that at 32 °C the vapor pressure of water (pv) is higher than at 25 °C. In order to take this into account, the permeability in Barrer units has been recalculated using Equation (4), (Table S7 in Supporting Information).

(4) $$P\;\left(\mathrm{Barrer}\right)=WVTR\frac{1441.3}{pv}$$

The water pressure of water, pv, is 3.6 cm Hg, so Equation (4) brings about a value of 2800 Barrer at 32 °C, which is actually 4 times the value obtained at 25 °C.

The enhancement of permeability is less remarkable in the case of the recycled PP/CNT sample that increases 2 times, probably because of the presence of TiO_2_ nanoparticles that hinder the permeation of water molecules.

Needless to say, our results indicate that applying different voltages to the membranes made of PP/CNT nanocomposites would provoke a temperature increase and, in turn, a permeability enhancement in consonance with the results shown in Figure 9.

## 4. Conclusions

All the investigated PP- and PE-based nanocomposites containing 5% CNT including those elaborated with recycled polymers, reached electrical conductivities above 10^−5^ S m^−1^. Best results are obtained with the PP/CNT nanocomposite, which reaches a conductivity of 0.1 S m^−1^ and taking advantage of the Joule effect, the temperature increases 90 °C when a voltage of 20 V is applied. Interestingly, for all the nanocomposites, the heating process was observed to be fast and reversible, since removing voltage lead rapidly to the initial temperature.

This outstanding stimulus (voltage)-response (temperature) feature was contemplated for its application to control transport properties, such as permeability, because the mobility of the polymer chains is enhanced, and the diffusion of the penetrant is facilitated as temperature is increased. Consequently, a route is shown to obtain non-porous membranes, which bring about a permeability regulated by the applied voltage.

## Figures and Tables

**Table 1:** Theoretical prediction of permeability.

Sample	Cussler random P O_2_ (Barrer)	Fredrickson–Bicerano P O_2_ (Barrer)	Experimental P O_2_ (Barrer)
PP/CNT	0.95	0.91	0.95
Rec-PP/CNT	1.99	1.65	1.39
PE/CNT	0.82	0.79	1.04
Rec-PE/CNT	1.13	0.94	1.12

## Supplementary Materials

The following are available online at https://www.mdpi.com/2073-4360/11/7/1180/s1, Figure S1: TEM micrographs of PE based nanocomposites, Figure S2: IR image of rec-PP; Figure S3: TGA of the rec-PP residue after carbonization, Figure S4: heating and cooling step of PE/CNT, Table S1: Parameters corresponding to the Joule effect for PP/CNT, Table S2: Parameters corresponding to the Joule effect for rec-PP/CNT, Table S3: Parameters corresponding to the Joule effect for PE/CNT, Table S4: crystallinity of membranes; Table S5: permeability of PP and PE at 24 °C, Table S6: Permeability predictions, Table S7: Permeability of PP and PE at different temperatures.
