# Supplementary material for: Elaboration and Characterization of Conductive Polymer Nanocomposites with Potential Use as Electrically Driven Membranes
Source: Polymers (Basel). 2019 Jul 13;11(7):1180. doi: 10.3390/polym11071180 (PMC6680706; doi:10.3390/polym11071180)
Supplement: Supplementary file 1 [file polymers-11-01180-s001.pdf]

Elaboration and characterization of conductive polymer nanocomposites with potential use as electrically driven membranes

Leire Sangroniz [1], Ainara Sangroniz [1], Mercedes Fernández [1], Agustin Etxeberria [1], Alejandro J. Müller [1,2] and Antxon Santamaria [1,*]

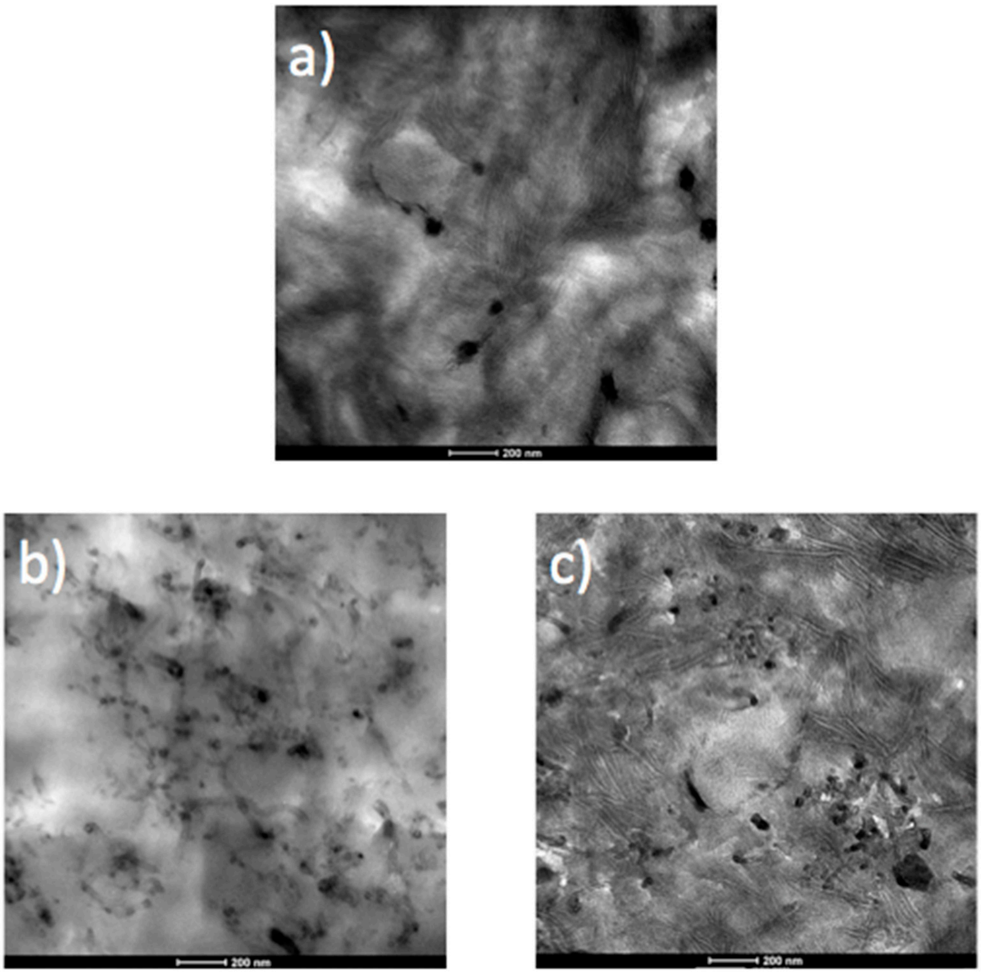

Figure S1. TEM images of the recycled PE and the different polymer/CNT nanocomposites. (**a**) Rec-PE, (b) PE/CNT and (c) Rec-PE/CNT.

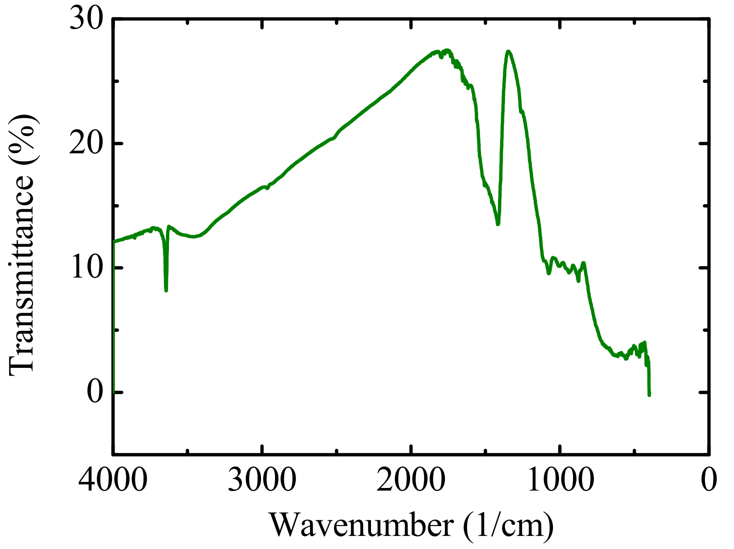

Figure S2. Infra red spectroscopy of Rec-PP sample residue after being carboniced.

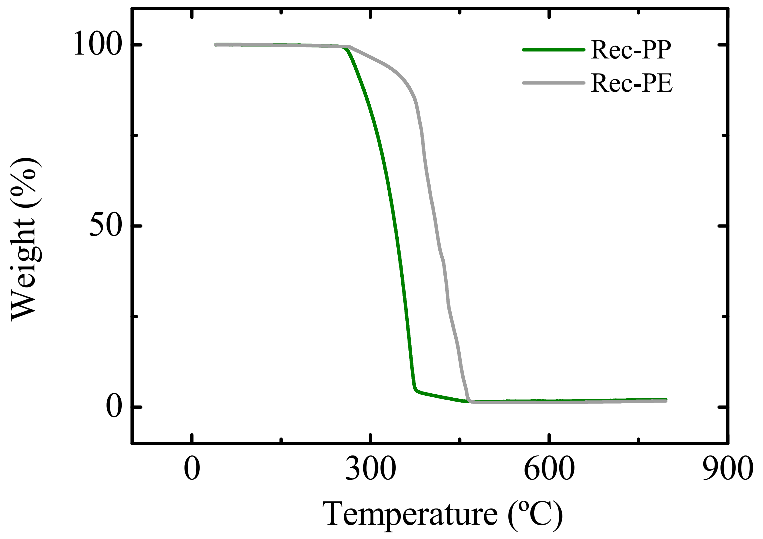

Figure S3. The weight loss obtained by TGA for recycled PP (Rec-PP) and recycled PE (Rec-PE).

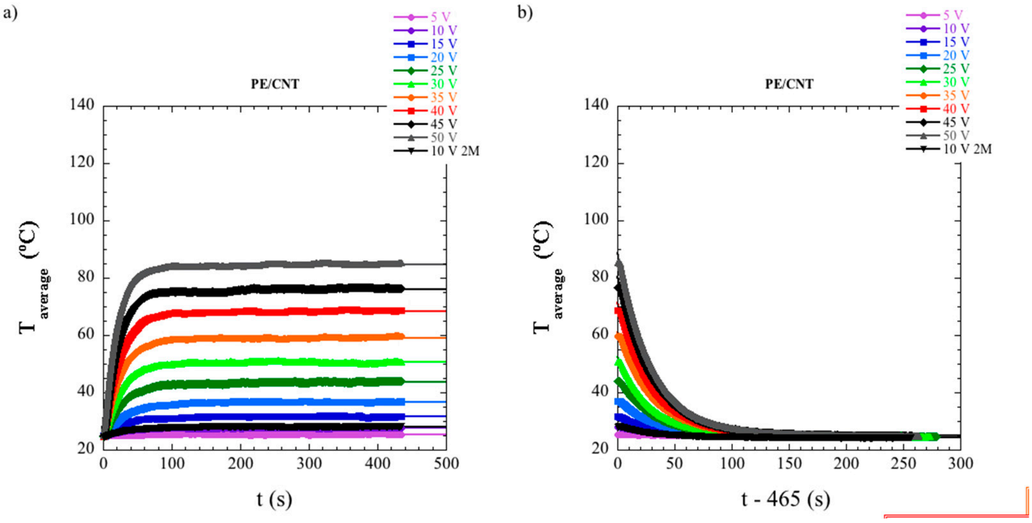

Figure S4. The heating and cooling step of the Joule heating effect is shown for PE/CNT. The data are fitted to equation 1 in the case of the heating step and to equation 3 for the cooling process. 10 V 2M stands for a second measurement carried out applying 10 V after a first run, to test reproducibility.

Table S1. Different parameters obtained using equation 1-3 for PP/CNT, h_{r+c} is the heat transferred by radiation and convection, τ_h and τ_c are a characteristic time, corresponding to heating and cooling respectively. C_p is the specific heat capacitance $\left(C_p = \frac{\tau}{R_{th} m}\text{, where m is the weigth}\right)$ and R_{th} is the thermal resistance ($R_{th} = \Delta T/P$). All the parameters have been obtained from the heating except τ_c. 10 V 2M stands for a second measurement carried out applying 10 V after a first run, to test reproducibility.

PP/CNT					
V	h_{r+c}	C_p	R_{th}	τ_h	τ_c
V	mW ºC^{-1}	J g^{-1} K^{-1}	ºC W^{-1}	s	s
2.5	34.0 ± 15.1	1.56 ± 0.29	117.0±56.2	37.1±10.7	41.5±12.0
5	37.6 ± 4.7	1.65 ± 0.19	94.4±17.4	32.8±1.6	30.6±2.2
7.5	37.1 ± 1.8	1.51 ± 0.01	93.7±1.6	30.1±1.2	30.7±0.7
10	33.2 ± 3.2	1.21 ± 0.20	108.0±10.6	27.8±2.4	29.8±0.6
12.5	38.1 ± 2.4	1.31 ± 0.16	92.2±8.0	25.7±1.4	30.2±1.1
15	34.5 ± 1.9	1.08 ± 0.14	102.2±9.2	23.3±1.4	29.4±1.1
17.5	36.5 ± 4.0	1.06 ± 0.07	97.1±8.8	21.9±0.9	29.0±0.2
20	39.6 ± 1.8	1.05 ± 0.11	88.8±6.6	19.8±1.0	29.1±0.1
10 2M	33.6 ± 3.9	1.14 ± 0.15	107.5±14.6	25.9±0.5	29.2±0.6

Table S2. Different parameters obtained using equation 1-3 for Rec-PP/CNT Powder, h_{r+c} is the heat transferred by radiation and convection, τ_h and τ_c are a characteristic time, corresponding to heating and cooling respectively. C_p is the specific heat capacitance $\left(C_p = \frac{\tau}{R_{th} m}\text{, where m is the weigth}\right)$ and R_{th} is the thermal resistance ($R_{th} = \Delta T/P$). All the parameters have been obtained from the heating except τ_c. 10 V 2M stands for a second measurement carried out applying 10 V after a first run, to test reproducibility.

Rec-PP/ CNT					
V	h_{r+c}	C_p	R_{th}	τ_h	τ_c
V	mW ºC^{-1}	J g^{-1} K^{-1}	ºC W^{-1}	s	s
5	601.4±422.4	34.31±15.11	8.4 ± 4.7	66.3 ± 59.2	138.6±164.1
10	748.5±727.5	85.25±90.82	8.3 ± 11.2	41.8 ± 39.6	48.7±16.3
15	149.2±21.0	9.26±1.87	23.8 ± 4.4	44.3 ± 0.9	37.9±10.4
20	101.8±1.6	5.58±0.14	34.8 ± 1.0	39.8 ± 2.1	38.3±1.2
25	73.1± 5.6	4.10±0.12	50.2 ± 5.5	42.0 ± 3.5	34.2±0.5
30	60.3±6.3	2.97±0.46	58.0 ± 5.8	35.0 ± 1.8	35.2±0.8
35	53.0±4.1	2.41±0.52	66.3 ± 4.6	32.4 ± 4.7	34.8±0.2
40	79.4±6.2	3.40±0.20	43.8 ± 3.0	30.4 ± 0.2	34.8±0.4
45	82.4±12.6	3.60±0.19	43.4 ± 6.7	31.8 ± 3.3	33.7±0.2
50	76.9±11.6	2.98±0.46	46.4 ± 7.0	28.0 ± 0.0	33.2±0.0
10 2M	180.9±16.3	10.20±3.27	18.7 ± 2.0	38.4 ± 8.3	38.2±0.7

Table S3. Different parameters obtained using equation 1-3 for PE/CNT, h_{r+c} is the heat transferred by radiation and convection, τ_h and τ_c are a characteristic time, corresponding to heating and cooling respectively. C_p is the specific heat capacitance $\left(C_p = \frac{\tau}{R_{th} m}\text{, where m is the weigth}\right)$ and R_{th} is the thermal resistance ($R_{th} = \Delta T/P$). All the parameters have been obtained from the heating except τ_c. 10 V 2M stands for a second measurement carried out applying 10 V after a first run, to test reproducibility.

PE/CNT					
V	h_{r+c}	C_p	R_{th}	τ_h	τ_c
V	mW ºC^{-1}	J g^{-1} K^{-1}	ºC W^{-1}	s	s
5	510.3±297.6	69.79±77.08	5.6±6.1	33.0±0.6	131.8±56.8
10	139.8±19.0	7.34±2.69	26.5±3.2	39.3±6.8	35.7±2.1
15	73.9±3.6	3.60±0.13	48.7±6.0	36.4±2.9	34.8±3.5
20	55.5±2.9	2.53±0.20	65.7±6.9	34.5±1.9	32.8±1.1
25	44.1±2.1	1.78±0.01	83.8±7.6	31.0±0.4	31.3±0.4
30	57.1±4.5	2.11±0.01	63.6±8.5	27.8±1.4	29.9±1.1
35	66.8±4.8	2.27±0.14	54.6±6.2	25.7±0.7	31.0±0.9
40	60.2±4.5	1.99±0.19	60.8±8.3	25.0±1.0	30.3±0.0
45	57.1±5.4	1.73±0.09	64.1±9.8	22.9±0.4	30.2±0.5
50	58.1	1.50	60.5	20.0	30.7
10 2M	100.6	4.90	35.5	38.4	36.5

Table S4. Crystallinity of membranes employed for permeality measurements.

Sample	X_C (%)
PP	51
Rec-PP	49
PP/CNT	49
Rec-PP/CNT	45
PE	75

Rec-PE	59
PE/CNT	70
Rec-PE/CNT	57

Table S5. Permeability of PP and PE systems at 25 °C.

Sample	WVTR ((g mm)/(m^2 day))	P O_2 (Barrer)
PP	2.67 ± 0.27	1.77 ± 0.20
Rec-PP	4.97 ± 0.36	3.09 ± 0.57
PP/CNT	0.95 ± 0.25	2.20 ± 0.09
Rec-PP/CNT	1.39 ± 0.27	3.18 ± 0.12
PE	2.32 ± 0.18	1.03 ± 0.04
Rec-PE	2.56 ± 0.32	1.48 ± 0.05
PE/CNT	1.04 ± 0.22	1.99 ± 0.06
Rec-PE/CNT	1.12 ± 0.16	2.45 ± 0.15

Table S6. Permeability prediction employing different models.

Sample	PP/CNT	Rec-PP/CNT	PE/CNT	Rec-PE/CNT
Nielsen	1.25	2.43	1.05	1.34
Cussler regular	1.36	2.86	1.18	1.63
Bharadwaj	1.79	3.22	1.55	1.83
Gusev Lusti	1.26	2.42	1.10	1.38

Table S7. Permeability of PP/CNT and Rec-PP/CNT at 25ºC and 32ºC.

Sample	WVTR ((g mm)/(m^2 day)) 25 °C	P H_2O (Barrer) 25 °C	WVTR ((g mm)/(m^2 day)) 32 °C	P H_2O (Barrer) 32 °C
PP/CNT	0.95 ± 0.25	575 ± 150	7.13 ± 0.95	2800 ± 340
Rec-PP/CNT	1.39 ± 0.27	840 ± 160	4.47 ± 0.51	1790 ± 270
